# Metallic plate in tobacco filters: A new pediatric challenge

**DOI:** 10.1002/jpn3.70132

**Published:** 2025-07-02

**Authors:** Annalisa Di Carmine, Marco Di Mitri, Cristian Bisanti, Edoardo Collautti, Simone D'Antonio, Michele Libri, Tommaso Gargano, Mario Lima

**Affiliations:** ^1^ Pediatric Surgery Department, IRCCS Sant'Orsola Alma Mater Studiorum University of Bologna Bologna Italy

**Keywords:** children, cigarettes, endoscopy, foreign body ingestion, sharp foreign body

## 1 INTRODUCTION

Foreign body (FB) ingestion is a common pediatric emergency, especially in younger children during the oral phase of their developmental behavior.^1^ This exploratory tendency is a natural phase of development, yet it places young children at a heightened risk of accidental ingestion of nonedible items. The ingestion of sharp objects is associated with significant risks, including mucosal trauma, gastrointestinal perforation, and in severe cases, life‐threatening complications requiring prompt intervention.^2^, ^3^ Pediatric FB ingestion accounts for a significant number of emergency department visits, with outcomes depending on the type of ingested object and the promptness of medical intervention.^4^ Recently, a new category of ingested foreign bodies has emerged: filters from tobacco products, specifically cigarette multicomponent filters containing metallic plates.^5^ While seemingly innocuous in their intended use, these objects present a unique clinical challenge due to their sharp edges.^6^ Unlike more common ingested objects such as coins or toys, these filters are designed with specific functional properties that unintentionally increase their risk profile when ingested by children. The growing availability of tobacco sticks with metallic elements highlights the need for increased awareness among clinicians and poison control centers about the risks of ingestion in pediatric populations. Tobacco products, particularly those designed with advanced filtration systems, have seen a surge in market popularity.^7^ This surge correlates with a notable increase in emergency cases involving pediatric ingestion of these filters. Filters containing metallic plates are integral to the functionality of certain consumer products, such as smoking devices, yet their unintended accessibility to children renders them a significant safety concern.^8^ Such ingestions frequently mandate urgent medical intervention, including endoscopic retrieval, to mitigate the risk of severe complications. The endoscopic approach is preferred due to its minimally invasive nature and effectiveness in removing foreign bodies without the need for surgical exploration. The rising prevalence of these incidents is associated with the growing popularity of products incorporating such filters, resulting in an uptick in pediatric emergency department visits for FB ingestion. This trend highlights the critical need for enhanced public awareness, preventive strategies, and informed clinical management. Targeted education for caregivers regarding the potential hazards of such products, alongside stricter regulatory measures to limit their accessibility, could significantly reduce the incidence of these events. The designation of these products as vulnerant by national health authorities reflects the escalating concern regarding their ingestion by children, necessitating vigilant pediatric care and early intervention.^9^ The present study aims to report and analyze cases of FB ingestion involving filters with a metallic plate over the last years. Specifically, it seeks to document the increasing incidence of these cases and provide a comparative analysis of the ingestion rates of these new filters versus other types of foreign bodies, examining clinical presentations, therapeutic interventions, and outcomes. Ultimately, the findings aim to foster improved preventive measures, enhanced safety standards, and reduced pediatric morbidity linked with these hazardous events.

## 2 METHODS

We conducted a case series study of ingestion of filters containing metallic plate from tobacco products which attended to our Pediatric Surgery Emergency Department of IRCCS Sant'Orsola‐Malpighi between January 1, 2023 and December 31, 2024. We analyzed the age distribution, clinical presentations, diagnostic work up, and endoscopic management.

### 2.1 Ethics statement

All procedures performed in studies involving human participants were in accordance with the ethical standards of the institutional and/or national research committee and with the 1964 Helsinki Declaration and its later amendments or comparable ethical standards. Ethical review and approval were waived for this study because it did not involve experimental procedures or interventions and because of the low number of patients involved.

## 3 RESULTS

We collected data from 89 pediatric patients who presented with FB ingestion between 2023 and 2024. Among these cases, foreign bodies were categorized as follows:

1. Nonvulnerant: 75 patients,
2. Vulnerant: 8 patients,
3. Button battery: 2 patients,
4. Food impaction: 4 patients.

**Figure 1:**
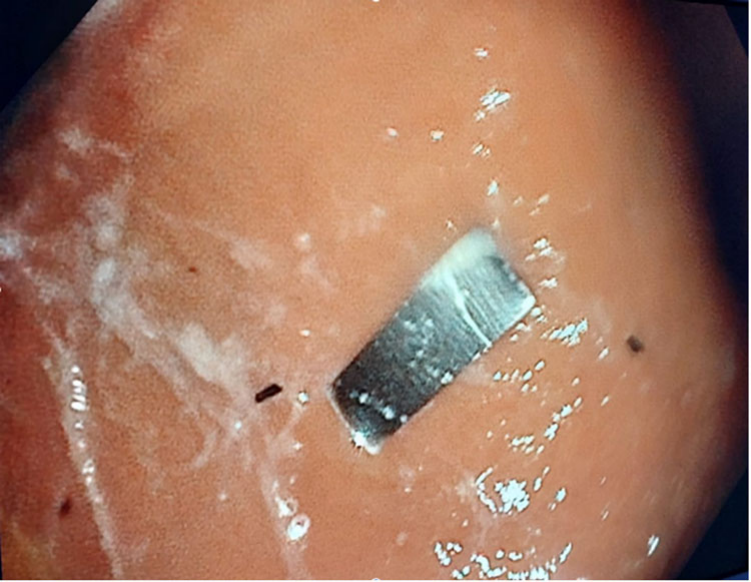
Metallic plate from tobacco product in the stomach during endoscopy.

Of the eight cases involving vulnerant foreign bodies, six were related to the ingestion of tobacco filters containing a metallic plate (Table 1). Among these six cases, two patients underwent urgent esophagogastroscopy due to the FB being in the stomach (Figure 1). The remaining four patients were managed conservatively under observation until the FB was naturally evacuated. The endoscopic removal of the FB was performed by an endoscopic basket to avoid any injuries. In two of the six cases, the patients had ingested only the metallic plate, which they had detached from the filter. Notably, no gastrointestinal injuries were documented in any of the cases, and all patients remained asymptomatic throughout their clinical course.

**Table 1:** Case series study: Characteristics and management of the patients.

Case	Age	Symptoms	X‐ray findings	Endoscopy	Complications
1	2 y.o.	None	X‐ray not performed	No	None
2	1 y.o.	None	FB in the stomach	No	None
3	2 y.o.	None	FB in the ileum	No	None
4	7 m.o.	None	FB in the ileum	No	None
5	11 m.o.	None	FB in the stomach	Successful removal	None
6	7 m.o.	None	FB in the stomach	Yes, FB not removed because pylorus passed	None

Abbreviations: FB, foreign body; m.o., months old; y.o., years old.

## 4 DISCUSSION

FB ingestion is one of the most common causes of emergency department admissions in the pediatric population. The management of FB ingestion can be challenging for physicians due to the wide variety of FB types (in terms of size and potential harm) and related symptoms, along with specific factors that must be considered in pediatric patients, such as age, underlying conditions related to intestinal dysmotility and known esophageal stenosis. Therefore, the management of these cases requires a certain level of experience and expertise, and physicians are advised to refer to clinical guidelines specifically tailored to the pediatric population.^10^

For foreign bodies with sharp or metallic components, such as the filters containing metallic plates described in this study, a structured management approach is essential to minimize the risk of gastrointestinal injury. In our case series, management decisions were based on established pediatric FB ingestion guidelines.^10^, ^11^ Key considerations included:

1. *Radiological evaluation*: all patients underwent an initial radiographic assessment to determine the FB's location. This was particularly crucial for metallic plate‐containing filters, as their radiopacity facilitated identification.
2. *Endoscopic versus conservative management*: in cases where the FB was located in the esophagus or in the an urgent esophagogastroscopy was performed for removal. If the object had migrated beyond the stomach and the patient remained asymptomatic, a conservative approach was followed, with close clinical observation and serial radiographs to confirm the passage.
3. *Symptoms‐based monitoring*: patients managed conservatively were observed for signs of complications, including abdominal pain, vomiting, or gastrointestinal bleeding, which would have necessitated urgent intervention.
4. *Follow‐up and counseling*: families were educated on the potential risks of re‐exposure, and preventive strategies were discussed, including safe storage of these products to minimize accidental ingestion (Figure S1).^12^

Although the potential risks and complications related to the exposure to tobacco and nicotine are generally known, as well as those associated with ingestion of e‐cigarette's liquid,^13^, ^14^ in recent years, a new category of ingested foreign bodies related to tobacco products has emerged: filters from heated tobacco products sticks. These devices include a 1‐cm metal heating element coated with stainless steel, designed to heat tobacco internally to over 350°C. Their sharp edges present a unique clinical challenge when ingested by children. The literature on the ingestion of these new types of foreign bodies is still very limited. Higashi et al.^15^ described a case about an asymptomatic 7‐month‐old girl who was admitted to emergency department after ingestion of heated tobacco filter, which was visualized in the stomach by X‐ray, so they decided to perform an endoscopic removal resulted only in slight esophageal mucosal abrasion with an uneventful postoperative course. Doi et al.^16^ reported two cases of natural excretion of metallic plate‐like filters from tobacco products after ingestion by an 11‐ and 9‐month‐old children without any complications. In our case series, we observed that the metallic plates ingested by children were generally small and smooth, which typically allows for safe passage through the gastrointestinal tract without intervention. This observation aligns with existing literature indicating that most ingested foreign bodies pass spontaneously without complications. However, when considering endoscopic retrieval, it's crucial to assess the potential risks associated with the procedure. The removal of metallic plates with sharp corners may pose a risk of injury to the esophagus or larynx, especially if appropriate protective measures are not in place. Therefore, the decision to proceed with endoscopic intervention should be carefully evaluated, weighing the risks of the procedure against the potential benefits. Moro et al.^17^ conducted an analysis of the data collected by the Milan Poison Control Center on the ingestion of metallic plate‐like filters between July 2023 and February 2024. Of the 40 patients involved, 16 exhibited symptoms, including repeated vomiting episodes and one tonsillar pillar and oral mucosa cut lesions, and two underwent endoscopic procedures. In recent years, there has been an increase in the consumption of heated tobacco stick and the growing availability of these products containing metallic elements underscores the necessity of heightened awareness among clinicians and poison control centers regarding the associated risks of ingestion by pediatric populations. Regarding the potential for nicotine exposure from these metallic plates, it's important to note that nicotine is highly toxic. However, the nonporous nature of the metallic material suggests that significant nicotine absorption is unlikely. Despite this, monitoring may be prudent, particularly in younger children who are more susceptible to toxins.

The packaging of these products contains a warning about sharp metallic components and the risk of serious injuries if ingested; indeed, sharp foreign bodies ingestion in pediatric patients can lead to severe risks, including mucosal trauma and severe gastrointestinal injuries,^18^ for this reason the retrieval performing endoscopic procedure is recommended. Concerning heated tobacco products, in our center, we opted for the removal of metallic plate‐like filter when located in regions accessible by endoscopy to mitigate the risk of serious complications. In the analysis of our case series, we observed that the most frequently ingested foreign sharp bodies over the past 2 years were filter from tobacco products with metallic plate‐like component. Considering our findings, a critical hypothesis emerges: the classification of these tobacco products as vulnerant might depend on the specific circumstances of ingestion. While the ingestion of the metallic plate detached from the filter could be reasonably classified as vulnerant due to its sharp edges and higher risk of causing injury, the ingestion of the metallic plate still encased within the filter appears to pose a significantly lower risk. The filter provides a protective barrier, reducing the likelihood of direct contact with mucosal surfaces and potential trauma. This suggests that the classification as vulnerant might not apply to cases where the metallic plate remains within the filter. Future research should focus on evaluating the differential risks of these scenarios to guide more precise regulatory and clinical protocols, ensuring both patient safety and appropriate risk categorization. Therefore, it is crucial to educate caregivers about the potential hazards of these products and limit their accessibility to children to reduce the risk of severe complications.

## 5 CONCLUSION

The increasing consumption of heated tobacco products with metal plate‐like filters has been linked to a rise in emergency department visits for FB ingestion, posing significant risks to children's health. This trend highlights the critical need for enhanced public awareness, preventive strategies, and informed clinical management to reduce the risk of critical complications.

## CONFLICT OF INTEREST STATEMENT

The authors declare no conflict of interest.

## Supporting information

Supplemental figure 1.

supmat.
