# Supplementary figures and images for: Metallic plate in tobacco filters: A new pediatric challenge
Source: J Pediatr Gastroenterol Nutr. 2025 Jul 2;81(3):748–52. doi: 10.1002/jpn3.70132 (PMC12408944; doi:10.1002/jpn3.70132)

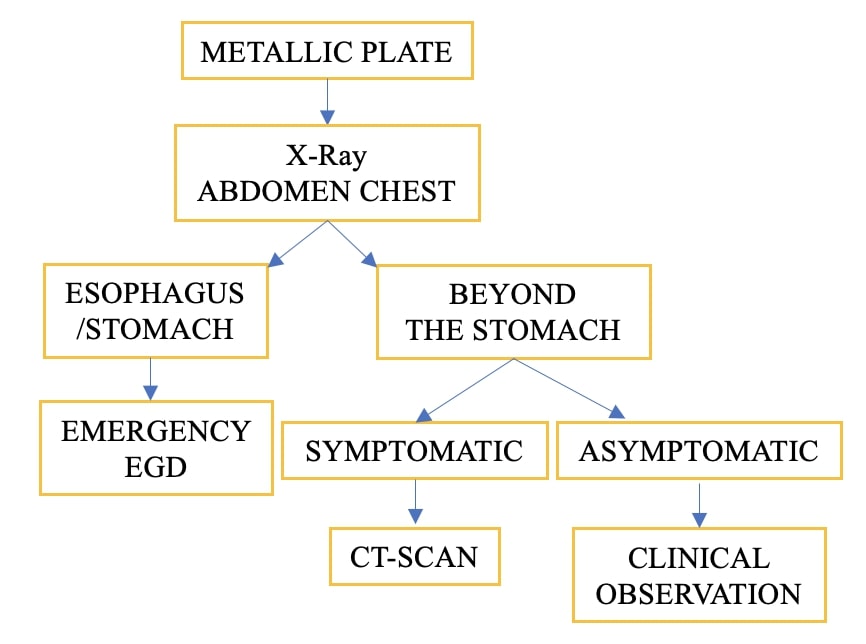

Supplement: Supplementary file 1 — Supplemental figure 1. [file JPN3-81-748-s001.jpg]
