# Supplementary material for: Metallic plate in tobacco filters: A new pediatric challenge
Source: J Pediatr Gastroenterol Nutr. 2025 Jul 2;81(3):748–52. doi: 10.1002/jpn3.70132 (PMC12408944; doi:10.1002/jpn3.70132)
Supplement: Supplementary file 2 — supmat. [file JPN3-81-748-s002.docx]

**Supplemental figure 1** - Management algorithm for pediatric foreign body ingestion of metallic plate contained in tobacco filters
